# Advancements in Micro-LED Performance through Nanomaterials and Nanostructures: A Review

**DOI:** 10.3390/nano14110940

**Published:** 2024-05-27

**Authors:** Aoqi Fang, Zaifa Du, Weiling Guo, Jixin Liu, Hao Xu, Penghao Tang, Jie Sun

**Affiliations:** 1 Key Laboratory of Optoelectronics Technology, Beijing University of Technology, Beijing 100124, China; fangaoqi@emails.bjut.edu.cn (A.F.);; 2 School of Physics and Electronic Information, Weifang University, Weifang 261061, China; 3 College of Physics and Information Engineering, Fuzhou University, Fuzhou 350100, China; 4 Fujian Science and Technology Innovation Laboratory for Optoelectronic Information of China, Fuzhou 350100, China; 5 Quantum Device Physics Laboratory, Department of Microtechnology and Nanoscience, Chalmers University of Technology, 41296 Gothenburg, Sweden

**Keywords:** micro-LED, quantum dot, nanomaterials, nanostructures

## Abstract

Micro-light-emitting diodes (μLEDs), with their advantages of high response speed, long lifespan, high brightness, and reliability, are widely regarded as the core of next-generation display technology. However, due to issues such as high manufacturing costs and low external quantum efficiency (EQE), μLEDs have not yet been truly commercialized. Additionally, the color conversion efficiency (CCE) of quantum dot (QD)-μLEDs is also a major obstacle to its practical application in the display industry. In this review, we systematically summarize the recent applications of nanomaterials and nanostructures in μLEDs and discuss the practical effects of these methods on enhancing the luminous efficiency of μLEDs and the color conversion efficiency of QD-μLEDs. Finally, the challenges and future prospects for the commercialization of μLEDs are proposed.

## 1. Introduction

LED devices with a mesa size smaller than 100 μm are referred to as μLEDs, which have become a hotspot in optoelectronic devices in recent years. Compared to mainstream displays like LCD and OLED, μLED offers many advantages, such as higher contrast, stronger reliability, and faster response speed [1]. In particular, with the continuous development of AR/VR in recent years, μLED is poised to become the core of next-generation display technology [2]. After two decades of technological accumulation, μLED has made significant breakthroughs in luminous brightness, size effects, color conversion, and other aspects [3], laying a technical foundation for its commercialization. According to TrendForce’s latest analysis [4], although the chip output value of µLEDs used in wearable devices, mainly smartwatches, was only USD 3 million in 2023, with Apple launching μLED-equipped smartwatches and the products hitting the market, the output value is expected to rapidly expand to USD 17.2 million and USD 46.3 million in 2024 and 2025, respectively. However, the maximum output value achievable by μLED display technology is far beyond this, and there are many factors constraining its rapid and large-scale application in the market. Factors such as epitaxial transfer [5,6,7], defect management [8,9], and bonding technology [10,11] leading to high manufacturing costs are the main reasons hindering its commercialization. Among them, the low external quantum efficiency (EQE) of μLEDs [12,13,14] and achieving full-color display [15,16,17,18] are among the main factors affecting its commercialization. In this paper, we discuss the main factors affecting the low EQE and luminous efficiency of μLED, as well as the low color conversion efficiency (CCE) of quantum dot (QD)-based μLEDs. We then discuss the improvements made in recent years in the structure and materials aimed at enhancing the luminous efficiency and CCE of μLEDs, mainly focusing on nanostructures and nanomaterials, such as nanorods, nanoring structures, and metal nanoparticles (NPs). We analyze the potential of nanostructures and nanomaterials to improve μLED performance from the perspective of experimental principles and material properties while also elucidating the limitations of these technologies and the challenges μLEDs currently face.

The low luminous efficiency and EQE of μLEDs are primarily attributed to the size effects [19]. In traditional large-sized LEDs, the sidewall area accounts for a small proportion of the overall emitting area, and sidewall defects have an insignificant impact on the overall performance. However, as the mesa size shrinks, sidewall effects become significant. Defects introduced during the etching process of the mesa surface will have a significant impact on the overall performance, leading to a sudden decrease in luminous efficiency [20,21]. The EQE of traditional blue LEDs can reach 80% [22], but in practical operation, if the size of such blue LEDs is reduced to 5–10 μm, the device’s EQE will be less than 20% [23]. Moreover, due to the presence of sidewall effects, the actual power consumption of current μLEDs is more severe than expected and is even higher than OLEDs [24,25]. In addition, there are many challenges associated with GaN-based μLEDs. Due to the high refractive index of GaN (refractive index of 2.5) [26,27], the critical angle for light escape is only 23°, which means that photons generated in the active region are likely to undergo total internal reflection at the exit interface, resulting in only 4% of the light energy being extracted [28]. Furthermore, the lattice mismatch between InGaN and GaN leads to significant strain within the material, resulting in high polarization fields and quantum-confined Stark effects (QCSEs) [29,30,31]. Red μLEDs are mainly composed of AlGaInP materials, and the increased defect density due to size effects leads to a more pronounced decrease in efficiency. Additionally, phosphide materials are highly susceptible to performance degradation due to thermal effects with increasing driving current [32,33]. Therefore, the efficiency of red μLEDs faces significant challenges, which is also a hindering factor for the full-colorization of μLEDs.

**Figure 1:**
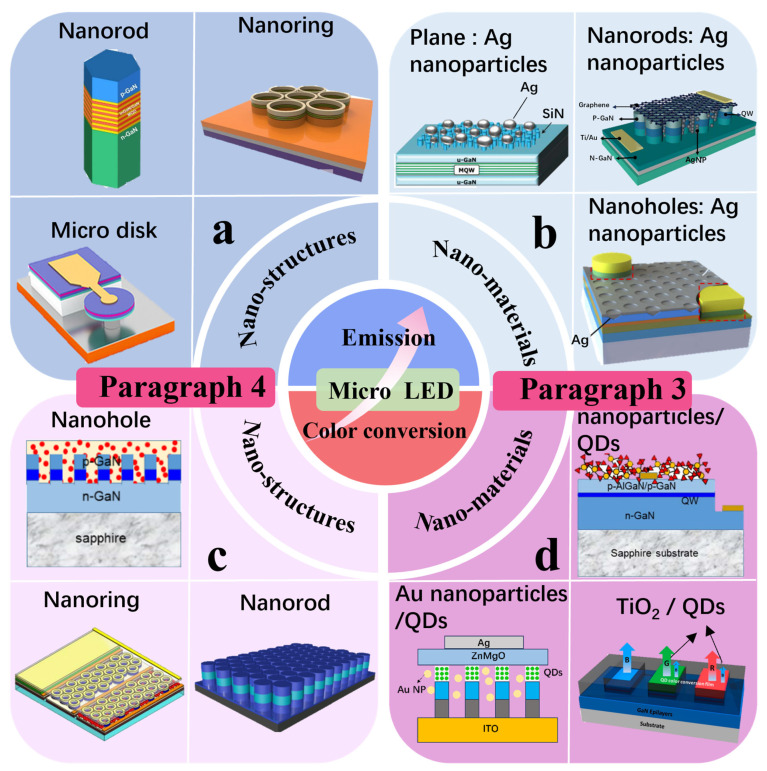
Schematic illustrating the strategies for improving the luminous efficiency and CCE of μLEDs using (**a**) nanostructures to enhance the luminous efficiency [34,35,36] (Reprinted with permission from ref. [34]. Copyright {2023} American Chemical Society. Reprinted with permission from ref. [35]. Copyright {2019} Nature/Scientific Reports. Reprinted with permission from ref. [36]. Copyright {2022} American Chemical Society), (**b**) nanomaterials to enhance the luminous efficiency [37,38,39] (Reprinted with permission from ref. [37]. Copyright {2009} Royal Society of Chemistry. Reprinted with permission from ref. [38]. Copyright {2022} IEEE. Reprinted with permission from ref. [39]. Copyright {2020} Optica Publishing Group), (**c**) nanostructures to improve the CCE [40,41,42] (Reprinted with permission from ref. [40]. Copyright {2022} Optica Publishing Group. Reprinted with permission from ref [41]. Copyright {2019} Photonic Research. Reprinted with permission from ref. [42]. Copyright {2023} IEEE), and (**d**) nanomaterials [43,44,45] to improve the CCE of μLEDs. Reprinted with permission from ref. [43]. Copyright {2019} Optica Publishing Group. Reprinted with permission from ref. [45]. Copyright {2021} American Chemical Society.

In response to the aforementioned issues, researchers have made significant efforts in improving device structures and introducing nanomaterials. This paper mainly focuses on enhancing the performance of GaN-based blue and green μLEDs using nanostructures and nanomaterials, while the improvement of red LED performance mainly focuses on the utilization of QD materials. Additionally, some QD-based nanostructures and nanomaterials are introduced to enhance the CCE and luminous efficiency of QD-μLEDs, replacing traditional red LEDs primarily composed of AlGaInP materials. In recent years, new structures applied to LEDs include nanorods, nanoholes, nanorings, etc., and the localized surface plasmon resonance (LSPR) coupling effect induced by metal NPs has also been introduced into research aimed at improving μLED luminous efficiency. The improvement of CCE for QD-based μLEDs is mainly based on non-radiative energy transfer (NRET) mechanisms and LSPR effects. We will discuss the efforts made by researchers in recent years to improve the luminous efficiency and CCE of μLEDs from the perspective of nanostructures and nanomaterials, as shown in Figure 1.

In the research on improving the luminous efficiency and color conversion efficiency of μLEDs using nanostructures and nanomaterials, many studies involve LSPR and NRET. Therefore, in this section, we introduce these two fundamental concepts.

## 2. Principle

### 2.1. Non-Radiative Energy Transfer (NRET)

In the traditional process of QD excitation in quantum well (QW) luminescence, light is emitted from the QW, irradiating the QDs. The QDs absorb the photon energy for radiative transitions and emit low-frequency photons. This energy transfer process through light excitation is termed radiative energy transfer. Due to total internal reflection and dispersion of photons during the escape process, the number of photons emitted to the external world is very small, and QDs cannot absorb all the incident photons when absorbing light. Hence, this process results in some waste, leading to a small effective utilization of photons during absorption conversion and, ultimately, low CCE. Therefore, the *NRET* mechanism is introduced into QD-LEDs to increase CCE [46,47,48,49,50].

**Figure 2:**
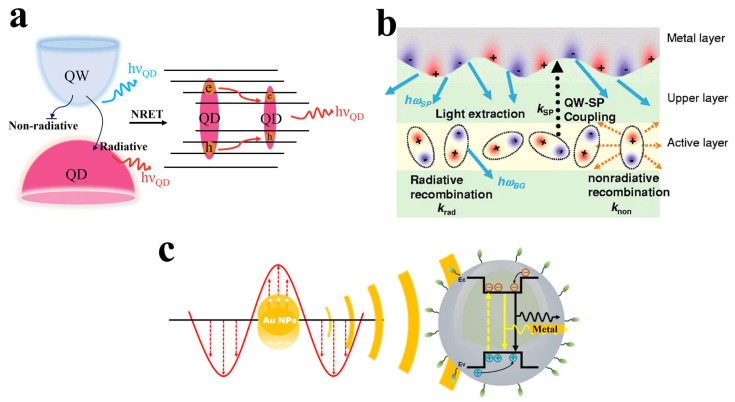
(**a**) Schematic diagram illustrating the principle of NRET [52]. (**b**) Schematic diagram illustrating the LSPR between metal NPs and QWs [56]. Reprinted with permission from ref. [56]. Copyright {2005} AIP Publishing. (**c**) Schematic diagram illustrating the LSPR between metal NPs and QDs [44]. Reprinted with permission from ref. [44]. Copyright {2024} American Chemical Society.

Figure 2a illustrates the basic process of *NRET* between QWs and QDs. The *NRET* process does not involve the absorption and emission of photons; instead, QDs directly absorb and relax the carrier energy from the QW within the band [51,52]. Thus, *NRET* reduces the absorption and re-emission of photons, significantly reducing the energy loss caused by these processes, thereby enhancing the color conversion efficiency and the quantum yield (QY) of QDs. However, *NRET* highly depends on the distance between QDs and the excitation light source, and the efficiency of energy transfer can be determined by the following equation [53,54,55]:

$$E_{NRET}=1/{\left[1+(r/r_{0}\right)}^{4}]$$

Here, ‘*r*’ represents the distance between the donor and acceptor, while ‘*r*_0_’ denotes the distance between the donor and acceptor when the energy transfer efficiency is 50%. For a specific system, the value of *r*_0_ remains constant. Therefore, shortening the distance between the QWs and QDs is crucial for enhancing the energy transfer rate and fully exploiting *NRET*.

### 2.2. Localized Surface Plasmon Resonance (LSPR)

When the size of metallic materials is much smaller than the wavelength of incident light, the electron cloud on the surface of the particles undergoes displacement relative to the atomic nucleus under the influence of the electric field of the light wave, forming localized plasmon resonances [56,57,58,59]. These resonances, matching the frequency of the incident light, result in a significant enhancement of the electromagnetic field on and near the metal surface. When the emitting materials (quantum wells and quantum dots) are close to metal NPs, their luminescence intensity and efficiency can be enhanced through LSPR, as illustrated in Figure 2b [44,57]. The electric field generated by localized surface plasmons (LSPs) is a transient field, decaying exponentially with increasing distance of field propagation [59,60]. In other words, the closer the material is to the light source, the stronger the coupling. Therefore, in μLEDs, shortening the distance between the QW and the metallic material is crucial for enhancing the efficiency of LSPR coupling.

## 3. Using Nanomaterials to Enhance μLED Luminous Efficiency and Color Conversion Efficiency

Introducing specific nanomaterials into LEDs can increase light scattering and enhance the light extraction efficiency of LEDs. Many researchers have placed materials such as metal NPs [61,62] or polystyrene [63,64] on the surface of LEDs to act as LSPs or scattering agents to improve the light-emitting performance of LEDs. QDs are also nanomaterials that can absorb high-frequency photons and emit low-frequency photons, playing an important role in the full colorization of μLEDs. In this section, we will mainly introduce the recent applications of some nanomaterials in LEDs, such as metal NPs, colloidal QDs, and TiO_2_ NPs.

### 3.1. Metal Nanoparticles (NPs)

#### 3.1.1. Using Ag NPs to Enhance Luminous Efficiency

Applying spin-coated Ag NPs on the surface and sidewalls of LEDs, matching the absorption resonance peak with the LED emission wavelength, and utilizing LSP resonance to enhance μLED luminous efficiency is a relatively simple method of using nanomaterials to improve device performance. In 2024, Sun et al. designed μLEDs of different sizes and spin coated Ag NPs on the top and sidewalls of these μLEDs [65]. Experimental results showed that for larger-sized devices, the electroluminescence (EL) intensity decreased after the addition of Ag NPs. This decrease was attributed to the fact that the Ag NPs on the surface of the μLED were too far away from the QW, exceeding 150 nm. This distance greatly exceeded the decay field range of LSPs; so, these Ag NPs only obstructed light emission and could not effectively couple with the emitting centers.

**Figure 3:**
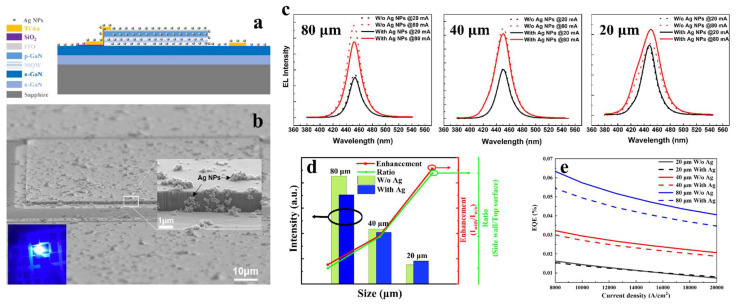
Enhancing GaN-based blue μLED luminous efficiency using Ag NPs. (**a**) Schematic diagram of the structure. (**b**) Scanning electron microscope (SEM) image of the device coated with Ag NPs. (**c**) Comparison of electroluminescence spectra of μLEDs with and without Ag NPs at different sizes under different currents. (**d**) Comparison of emission intensity with and without (W/o) Ag NPs at injection current of 80 mA and the dependence of enhancement (I_with_/I_without_) on sidewall proportion at different μLED sizes. (**e**) Variation of EQE at different current densities for three different samples under different current densities [65]. Reprinted with permission from ref. [65]. Copyright {2024} Springer Nature.

Meanwhile, in larger-sized devices, the proportion of sidewall area is relatively small, and the enhancement of luminous efficiency brought by LSP coupling at the sidewalls is not enough to compensate for the loss of light extraction caused by surface coverage. Therefore, there is a noticeable decrease in EL intensity. As the size of the μLED continues to shrink, the proportion of sidewall emission gradually increases, and the emitting area on the top surface gradually decreases. The benefits of suppressing non-radiative recombination through localized surface plasmon coupling slowly outweigh the reduction in light extraction caused by surface coverage. This is manifested in the increase in electroluminescence intensity. As shown in Figure 3d,e, as the mesa size decreases, the enhancement effect of LSPs on device emission intensity and EQE becomes more pronounced. In μLEDs with a mesa size of 20 × 20 μm^2^, compared to μLED samples without LSP coupling, EQE increased by approximately 8% under a high current density of 20,000 A/cm^2^. This study provides a valuable reference for the application of LSPs in enhancing the luminous performance of μLEDs.

#### 3.1.2. Using Ag NPs to Enhance Color Conversion Efficiency

**Figure 4:**
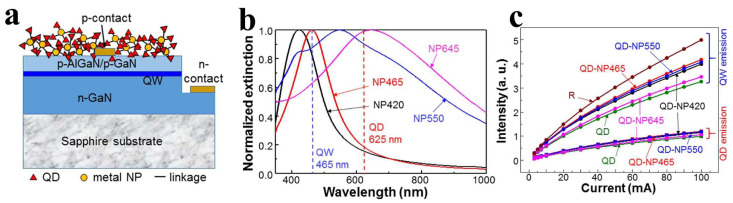
(**a**) Schematic diagram of the device structure. (**b**) Absorption spectra of different metal NPs, with the numbers in the metal NP names indicating the absorption spectrum peak values. (**c**) Variation of QW and QD emission intensities with injection current for different LED samples [43]. Reprinted with permission from ref. [43]. Copyright {2019} Optica Publishing Group.

Enhancing the absorption efficiency of QDs for the excitation light source is a key aspect of applying QDs in full-color displays using μLEDs. Mixing metal NPs with QDs can enhance the energy conversion efficiency of QDs. In 2019, the Yang group coated different types and sizes of metal NPs mixed with QDs on the LED surface [43]; the absorption spectra of different types of metal NPs are shown in Figure 4b. The research results showed that Ag NPs, whose absorption resonance peak matches the QW emission wavelength, can enhance the emission intensity of QWs through QW-LSP coupling, thereby enhancing the emission intensity of QDs, as shown in Figure 4c.

The time-resolved photoluminescence (TRPL) of different devices is shown in Table 1, indicating that Ag NPs with absorption resonance peaks matching the QW emission wavelength can simultaneously increase the carrier decay rates of both QWs and QDs, leading to an overall improvement in energy conversion efficiency. This study fully demonstrates the positive role of LSP resonance in the emission of QDs. However, this experiment requires a sufficiently thin P-GaN layer to meet the requirements of LSP resonance for distance, which may have a significant impact on the performance of the LED [66].

### 3.2. TiO_2_ Nanoparticles

**Figure 5:**
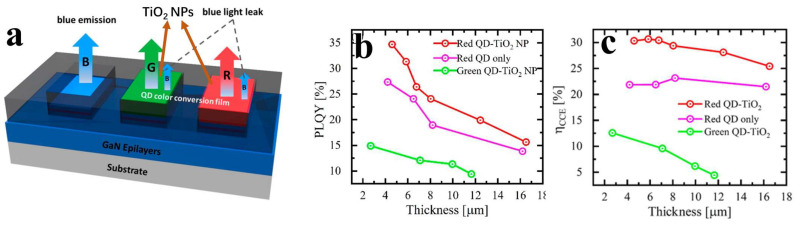
(**a**) Schematic diagram of the device structure. Variation of (**b**) QY and (**c**) CCE of red and green QDs with QD thickness after the addition of TiO_2_ NPs [45]. Reprinted with permission from ref. [42]. Copyright {2021} American Chemical Society.

In 2021, Kuo’s group added TiO_2_ NPs to red and green QDs [45], as shown in Figure 5a. Unlike LSPR, TiO_2_ enhances the scattering effect [67]; so, there is no need to deliberately thin the P-GaN layer. The experimental results showed that the scattering effect of TiO_2_ NPs enhanced the luminous intensity by more than 10%. Figure 5b shows the QY of two types of QDs, indicating a significant improvement in the QY of QDs after adding TiO_2_. The same change is also reflected in Figure 5c. This study suggests that the combination of QDs with other nanomaterials may enhance their QY and achieve better color conversion effects.

Numerous research results indicate that nanomaterials such as Ag NPs, polystyrene, metal oxide NPs, and others have a positive impact on enhancing the light-emitting performance of LEDs [68,69,70,71]. However, constrained by the LED’s own structure, these nanomaterials may not fully exert their maximum potential. Therefore, combining nanostructures with nanomaterials will provide greater assistance in improving the luminous efficiency and CCE of LEDs.

## 4. Using Nanostructures to Enhance μLED Luminous Efficiency and Color Conversion Efficiency

The nanostructures discussed in this review include nanorods, nanoholes, and nanorings. Nanorod structures can be formed by self-assembling nanospheres as etching hard masks or by molecular beam epitaxy (MBE). Nanohole structures are formed by a combination of nanoimprinting and inductively coupled plasma reactive ion etching (ICP-RIE). Nanoring structures can be prepared by using self-assembled nanospheres as etching hard masks or by a combination of electron beam lithography and ICP-RIE. Detailed fabrication processes are described in the following sections.

### 4.1. Nanoring Structures

#### 4.1.1. Using Nanorings to Enhance Luminous Efficiency

**Figure 6:**
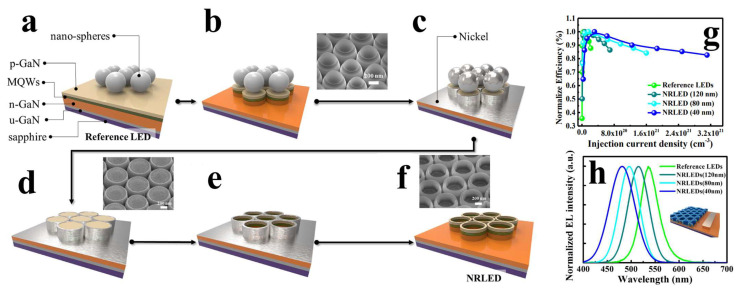
(**a**) Uniform deposition of polystyrene (PS) nanospheres on the LED substrate. (**b**) Generation of nanorod arrays through the ICP-RIE process. The inset shows the SEM image of the nanorod LEDs. (**c**) Reduction of the diameter of the nanospheres through O_2_ plasma treatment, followed by deposition of nickel metal onto the nanorods of the LED. (**d**) Nanorod LEDs coated with nickel metal as a protective layer. The inset displays the SEM image of nickel-coated nanorod LEDs. (**e**) Fabrication of the nanoring LED template after the ICP-RIE etching process. (**f**) Removal of nickel using acidic solution to obtain the nanoring LED. The inset shows the SEM image of the nanoring LED. The (**g**) EQE and (**h**) EL spectra of nanoring LEDs of different sizes compared to the reference LED (conventional LED) [35]. Reprinted with permission from ref. [35]. Copyright {2019} Nature/Scientific Reports.

Introducing nanostructures into InGaN/GaN multiple quantum wells (MQWs) can significantly reduce the material’s strain relaxation, thereby improving QCSEs. Applying this concept to μLEDs can enhance the device’s luminous efficiency. Additionally, due to changes in the internal polarization field of the QWs, the emission wavelength of the QWs can be blueshifted. Precise control of the nanostructure size allows for accurate modulation of the emission wavelength range [29,72,73,74,75,76,77,78]. Based on this concept, in 2017, the Kuo research group fabricated nanoring arrays on LED epitaxial wafers by depositing nickel on the surface and etching out nanorings with an outer diameter of 800 nm and an inner diameter of approximately 700 nm [35], the fabrication process is illustrated in Figure 6a–f. The nanoring structure improved the strain inside the material, resulting in nanoring LEDs with higher internal quantum efficiency (IQE) than traditional LEDs. More importantly, by adjusting the width of the nanorings, they precisely modulated the emission wavelength of the nanoring LEDs and achieved emission in four different colors on the same epitaxial wafer. This successful practice not only addresses the decrease in IQE caused by improving the LED epitaxial internal polarization field but also provides new insights for achieving full-color μLEDs.

#### 4.1.2. Using Nanorings to Enhance Color Conversion Efficiency

**Figure 7:**
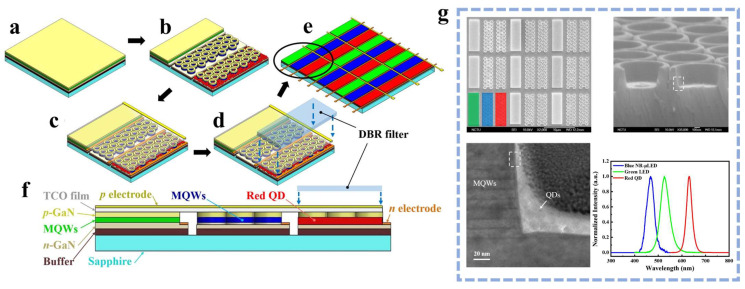
(**a**) Epitaxial wafer. (**b**) Three sub-pixels of a green μLED, a blue nanoring-μLED, and a red QD-nanoring-μLED. (**c**) Deposition of TCO film and pn electrodes. (**d**) Covering the DBR(distributed Bragg reflector) filter. (**e**) Full-color display panel composed of the proposed hybrid QD-nanoring-μLEDs. (**f**) Cross-sectional view of a single RGB pixel. (**g**) SEM image of RGB pixel array (top view), nanoring-μLED with 30° tilt angle, transmission electron microscope (TEM) image of the contact area between MQWs and QDs, and EL spectra of RGB hybrid QD-nanoring-μLEDs [41]. Reprinted with permission from ref. [41]. Copyright {2019} Photonic Research.

Based on the nanoring structure, LED emission wavelength can be precisely controlled, and the nanoring structure has significant advantages in shortening the distance between QDs and QWs. In 2019, Kuo’s research group etched nanoring arrays on GaN-based green LED epitaxial wafers and achieved single-chip integration of RGB μLEDs by adding red QDs to blue nanoring LEDs [41]. Figure 7a–f illustrates the process of achieving RGB using nanoring LEDs and QDs. In the experiment, the distance between the nanorings and QDs was small enough (as shown in Figure 7g), allowing full utilization of the advantages of NRET. This approach not only achieved full colorization but also enhanced the CCE, as demonstrated by the EL spectra of RGB sub-pixels shown in Figure 7g. It can be seen that combining nanoring structures with QDs effectively enables single-chip RGB emission on a homogeneous substrate.

Although nanoring structures can effectively alleviate internal strain and suppress QCSEs, their combination with QDs enables RGB emission in μLEDs. However, nanoring structures sacrifice a considerable amount of active area, resulting in low light intensity, and their fabrication process is relatively complex, making them difficult to apply to large-area full-color displays. These are the main factors constraining their further development [9].

### 4.2. Nanohole Structure

#### 4.2.1. Using Nanoholes to Enhance Luminous Efficiency

Shortening the distance between QDs or metal NPs and MQWs is a primary approach for the application of NRET and LSPR in LEDs. Etching nanohole arrays on the LED surface to the active region allows for effective contact between QDs or metal NPs and MQWs, and the introduction of nanoholes enhances emission through the cavity effect [49,79,80,81]. Moreover, these nanohole arrays do not affect the conductivity of the P-GaN surface and can be used to prepare metal electrodes using traditional methods. In this section, we will mainly discuss the combination of nanohole arrays and nanomaterials to enhance the light emission efficiency and CCE of μLEDs.

**Figure 8:**
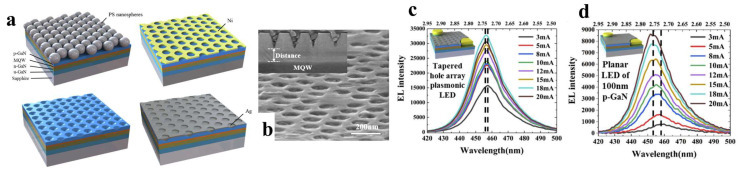
(**a**) Device fabrication process. (**b**) SEM images of nanoholes filled with Ag NPs. (**c**,**d**) EL spectra of nanohole LEDs with Ag NPs and planar LEDs [39]. Reprinted with permission from ref. [39]. Copyright {2020} Optica Publishing Group.

In 2020, Yun et al. fabricated plasmon-enhanced LEDs with conical nanohole structures [39], as illustrated in Figure 8a. Figure 8b shows the surface and cross-sectional SEM images of the device, indicating that the conical nanohole structure shortened the distance between Ag and the MQW, significantly improving the plasmon coupling efficiency. Figure 8c,d displays the EL spectra of planar LEDs and conical-nanohole LSP-enhanced LEDs. Compared to traditional planar LEDs, the EL intensity increased by 16 times. The enhancement in long-range coupling efficiency stems from the accumulation of LSP energy at the apex of the metal cone and momentum loss provided by the ripple surface. Therefore, even with a thick p-GaN layer, LSP-enhanced LEDs with conical Ag structures can maintain high light emission efficiency. Additionally, due to the compensation of the polarization-induced electric field in the MQW by QW-LSP coupling, the efficiency of carrier recombination in the MQW is enhanced [82,83], and the reduction in QCSEs resulting from the decrease in internal stress in the LED material [84,85] significantly reduces the blue shift of the EL emission peak in LSP-enhanced LEDs. Consequently, with increasing current, LSP-enhanced LEDs with conical Ag structures can maintain higher light emission efficiency and a more stable operating state.

**Figure 9:**
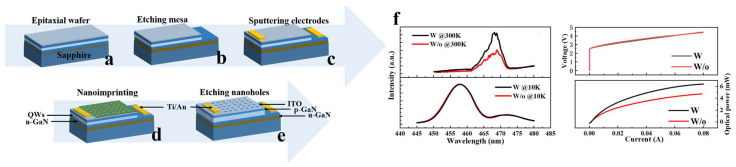
(**a**–**e**) Device fabrication process. (**f**) PL spectra, IV characteristics, and optical power of nh-μLED s with and without Ag NPs filling [86]. Reprinted with permission from ref. [86]. Copyright {2020} IOP Publishing.

In 2022, the Guo and Sun research groups achieved surface plasmon-enhanced nanohole μLEDs (NH-μLED) using nanoimprint lithography [86]. The fabrication process is illustrated in Figure 9a–e. In contrast to the conical nanohole structures mentioned above, they etched nanoholes into the N-GaN layer. This deep nanohole structure allows direct contact between Ag and the MQW, thereby achieving more effective LSP–QWs coupling. The optical and electrical properties of the device are shown in Figure 9f. The IQE of NH-μLEDs filled with silver NPs increased by 12%, resulting in an overall enhancement in light output power.

#### 4.2.2. Using Nanoholes to Enhance Color Conversion Efficiency

**Figure 10:**
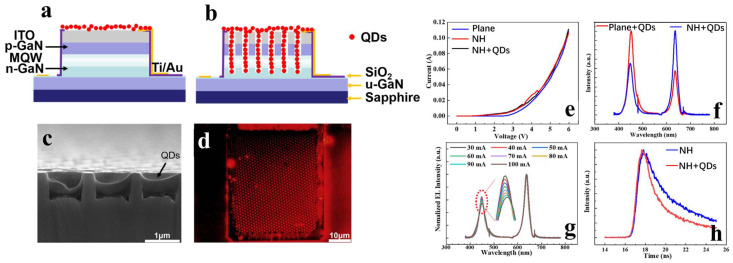
(**a**,**b**) Planar QD-μLED and NH-QD-μLED. (**c**) Cross-sectional SEM image of the NH-QD-μLED. (**d**) Fluorescence microscopy image of the NH-QD-μLED. (**e**) IV characteristics of the device. (**f**) Comparison of EL spectra between planar and NH-QD-μLEDs. (**g**) Transient current spectra of the NH-QD-μLED. (**h**) TRPL of NH-μLED and NH-QD-μLED [87]. Reprinted with permission from ref. [87]. Copyright {2021} IEEE.

Considering that NRET also heavily depends on the distance between QDs and QWs, the research group combined the nanohole structure with QDs to fabricate NH-QD-μLEDs [87]. Compared to conventional planar QD-μLEDs where QDs are spin coated on the surface (Figure 10a), the NH-QD-μLEDs (Figure 10b) exhibit similar electrical properties but demonstrate superior color conversion characteristics, with a CCE improvement of 118%. Additionally, as shown by the TRPL data in Figure 10g, the introduction of QDs into the nanoholes leads to a significant decrease in the carrier lifetime of the QWs. However, the deposition of QDs does not alter the intrinsic carrier dynamics of the QWs, indicating that the NH + QD structure provides an additional relaxation pathway for carriers in the QW, namely NRET.

**Figure 11:**
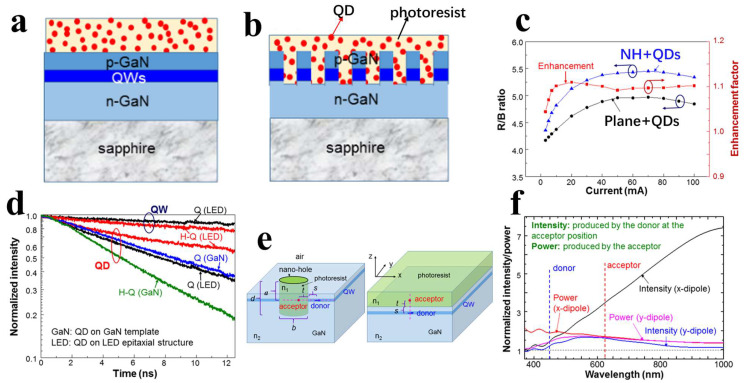
(**a**) Planar structure. (**b**) Nanohole structure. (**c**) Enhancement effect of the nanohole structure on the ratio of red to blue light intensity in EL spectra. (**d**) TRPL of QWs and QDs in planar and nanohole structures. (**e**) Simulation Model of Nanoporous Structure. (**f**) Simulated spectra of the normalized field intensity produced by the donor at the acceptor position and the normalized radiated power produced by the acceptor for the structure shown in Figure 11e. [40]. Reprinted with permission from ref. [40]. Copyright {2022} Optica Publishing Group.

In 2022, Yang’s research group utilized photolithography to encapsulate QDs within nanoholes, enabling the use of lithography techniques to design spatial QD distribution patterns on LED samples [40]; the planar structure and nanohole structure are shown in Figure 11a and Figure 11b, respectively. In addition to enhancing the efficiency of NRET between QWs and QDs, the nanohole structure introduced a nanoscale cavity effect representing a near-field Purcell effect. Through TRPL studies on QDs inserted into nanoholes within undoped GaN template structures, it was found that the emission efficiency of the inserted QDs significantly increased due to the nanoscale cavity effect. Figure 11e,f depicts the simulation results of the nanohole cavity effect, indicating a significant enhancement in the emission intensity of the emitting source with the nanohole structure, and this effect could also enhance NRET efficiency, especially for radiative dipoles in QWs perpendicular to the nanohole sidewalls. This research suggests that introducing nanostructures in LEDs not only enhances NRET efficiency but also modifies the radiative behavior of QDs through the Purcell effect [79], making their far-field emission stronger.

**Figure 12:**
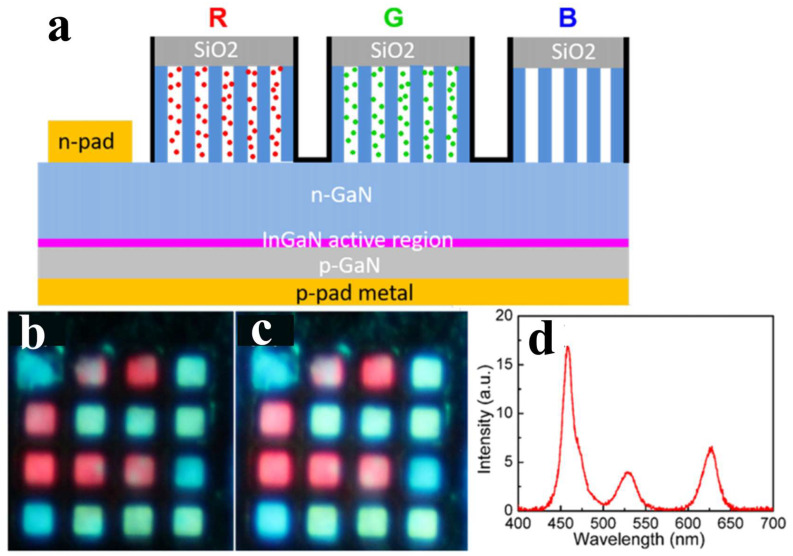
(**a**) Schematic diagram of the structure. (**b**,**c**) RGB μLEDs excited at injection currents of (**b**) 5 and (**c**) 7 mA. The size of each sub-pixel is about 35 × 35 μm^2^. (**d**) EL spectra of RGB μLEDs under a 5-mA current [88]. Reprinted with permission from ref. [88]. Copyright {2021} American Chemical Society.

Incorporating nanohole structures into full-color displays can also demonstrate excellent performance. In 2021, Song et al. embedded multi-color QDs in nanoholes to achieve different emission colors and manufactured RGB μLED arrays with pixel sizes of approximately 35 × 35 μm^2^ [88]; the structure is shown in Figure 12a. Although the nanoholes were not etched into the active region, thus not inducing NRET between QWs and QDs, the nanoholes could induce strong light scattering effects, significantly increasing the optical transmission path for blue light [89,90]. This enhanced absorption and re-emission within the nanoholes, resulting in CCEs of 98% and 63% for the embedded red and green QDs, respectively.

**Figure 13:**
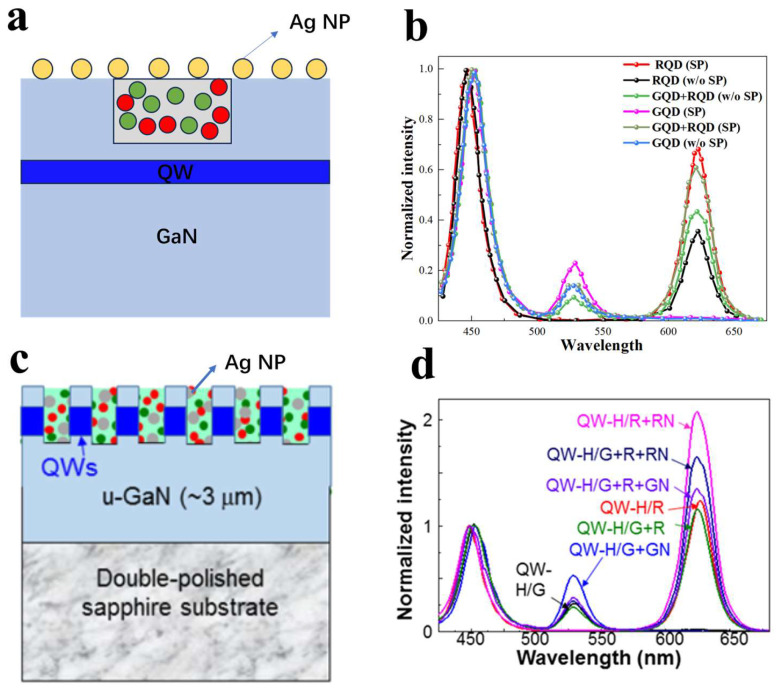
Ag NPs on the surface and QDs in the nanoholes (**a**) Schematic diagram. (**b**) EL spectra. Both QDs and Ag NPs are simultaneously filled into the nanoholes [92]. (**c**) Schematic diagram. (**d**) EL spectra, where “QW-H” represents the structure with nanoholes, “R” and “G” denote red and green Ds, respectively, and “RN” and “GN” represent Ag NPs whose absorption resonance peaks match the emission wavelengths of red and green QDs, respectively [93]. Reprinted with permission from ref. [93]. Copyright {2021} Optica Publishing Group.

If both LSPR and NRET can be utilized simultaneously, it would provide greater assistance in enhancing the performance of LEDs [91]. Yang’s research group designed two structures and characterized them using PL. The first structure, as shown in Figure 13a, involves nanoholes etched to a depth not exceeding the thickness of the P-GaN layer, filled with red and green QDs and covered with a layer of Ag serving as LSP [92]. PL testing (shown in Figure 13b) indicates that both red and green PL intensities are enhanced by the SP coupling, clearly demonstrating the enhanced color conversion from QWs to QDs. Furthermore, the research group etched nanoholes into the N-GaN layer and filled them with QDs and Ag (shown in Figure 13c) [93], allowing for closer contact and coupling between QWs, QDs, and LSPs. The PL spectra of different samples, as shown in Figure 13d, indicate that when only one type of QD is present, the LSP matching the emission wavelength of the current QD significantly enhances its emission intensity. When both red and green QDs are present, the LSP matching the emission wavelength of the red QD contributes more prominently to the overall CCE.

### 4.3. Nanorod Structure

#### 4.3.1. Using Nanorods to Enhance Luminous Efficiency

The nanorod structure can also play a similar role as nanoholes and nanorings. Moreover, compared to other nanostructures, nanorod structures can save more active area. Additionally, nanorods provide more escape paths for photons, similar to the effect of photonic crystals, thereby the enhancing light extraction efficiency of LEDs [94,95,96,97]. In recent years, many researchers have combined nanomaterials with nanorod structures to enhance the performance of μLEDs.

**Figure 14:**
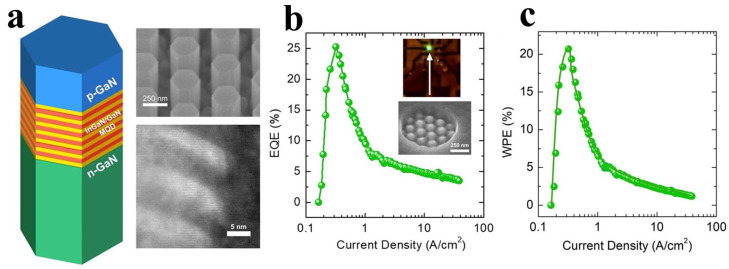
(**a**) Schematic diagram of nanorod structure, SEM image of nanorod array, and TEM image of MQW. (**b**) EQE and (**c**) WPE measured at different current densities for nanorod device [34]. Reprinted with permission from ref. [34]. Copyright {2023} American Chemical Society.

In 2023, the Mi research group demonstrated a submicron-sized green LED, and based on this, fabricated a μLED based on a nanorod array [34]. The individual nanorod structure and nanorod array are shown in Figure 14a, Figure 14b and Figure 14c demonstrate their EQE and wall-plug efficiency (WPE), which are 25.2% and 20.7%, respectively. The enhanced performance of the LED in this study is attributed to the nanorod structure achieving strain relaxation, utilizing semi-polar planes to minimize polarization effects and forming nanoscale quantum confinement to enhance electron–hole wavefunction overlap.

The combination of nanorod structures with nanomaterials can significantly enhance the light emission efficiency of μLEDs. Guo and Sun’s research groups achieved improvements in both the light emission efficiency and CCE of μLEDs by integrating nanorod structures with Ag NPs [38] and QDs [42], respectively.

**Figure 15:**
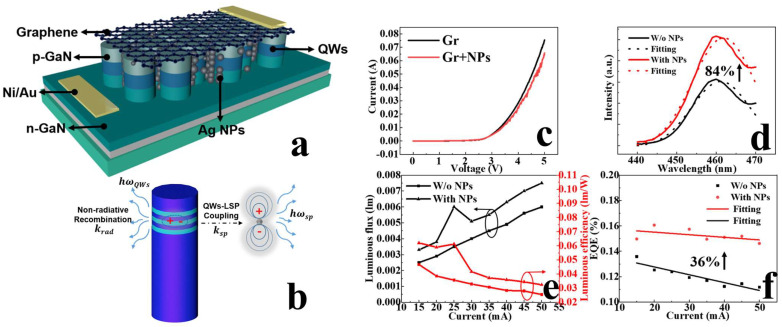
(**a**) Device structure schematic. (**b**) Schematic of LSPR between nanorods and Ag NPs. (**c**–**f**) IV characteristics, PL spectra, luminous flux, luminous efficiency, and EQE of devices with and without Ag NPs [38]. Reprinted with permission from ref. [38]. Copyright {2022} IEEE.

The research groups filled the gaps between nanorods with Ag NPs, ensuring full contact between Ag NPs and the sidewalls of nanorods. They then transferred a layer of graphene as a transparent conductive layer to connect independent nanorods, thereby utilizing the coupling between LSPs and QWs and the high conductivity and high transmittance of graphene to prepare an LSP-enhanced μLED [38]. Figure 15a illustrates the device structure, while Figure 15b depicts the schematic of the coupling between nanorods and LSPR. At room temperature, the PL intensity of the nanorod-µLED structure assisted by LSPs (before graphene transfer) increased by approximately 84%, and the EQE of the nanorod-μLED after graphene transfer remained 36% higher. This study applied LSPs and graphene to nanorod LEDs, enhancing the light emission efficiency of μLEDs and addressing the issue of non-contiguous nanorods, thereby opening up prospects for the application of LSPs in nanorod LEDs.

#### 4.3.2. Using Nanorods to Enhance Color Conversion Efficiency

**Figure 16:**
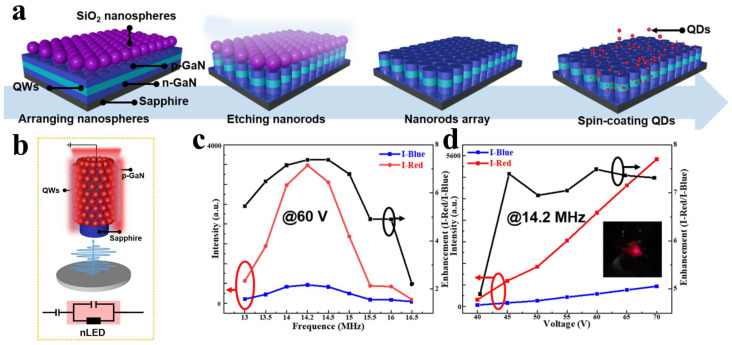
(**a**) Schematic of the fabrication process of QD-NLED. (**b**) Schematic diagram of a single nanorod and the driving circuit. (**c**) I-Red, I-Blue, and the ratio of I-Red to I-Blue under 60-V voltage. (**d**) I-Red, I-Blue, and the ratio of I-Red to I-Blue under 14.2-MHz frequency. The inset shows the emission image of nLED at 14.2 MHz and 60 V [42]. Reprinted with permission from ref. [42]. Copyright {2023} IEEE.

The combination of nanostructures and nanomaterials not only enhances the luminous efficiency of μLEDs, but also enables efficient color conversion when combined with QDs [98]. Figure 16a illustrates the fabrication process of the device. In this study, silica nanospheres were utilized as self-assembled masks for etching, resulting in the formation of uniformly distributed nanorods. Subsequently, QDs were filled into the nanorods, achieving ultra-high color conversion efficiency through NRET [42]. The schematic diagram of the driving circuit for QD-NR structure is shown in Figure 16b, unlike traditional direct current driving methods, a single-side contact alternating current driving method was adopted, eliminating electrode growth and simplifying the fabrication process. Test results demonstrated that maximum electroluminescent intensity and CCE were achieved when applying an alternating current with a frequency of 14.2 MHz, as shown in Figure 16c and Figure 16d. Under the conditions of 60 V and 14.2 MHz, the color conversion efficiency of the QD-based nanorod LED (nLED) used in this work was approximately 86.67%. This research confirms that the nanorod structure exposes more QWs for contact with QDs, thereby enabling the NRET mechanism between QDs and QWs to play a significant role in color conversion for μLEDs. Furthermore, the single-side contact alternating current driving method adopted in this experiment simplifies the fabrication process of μLEDs. Through the principle of special carrier injection, it can improve the crosstalk between μLED pixels and enhance the luminous efficiency of μLEDs [99,100,101,102].

Although nanorod arrays can release stress to the maximum extent and increase LEE by sacrificing fewer active regions and combining them with certain nanomaterials can enhance LED performance, the discontinuity between nanorod arrays presents challenges for current spreading and electrode fabrication. Single-side contact alternating current driving is not practical for actual production. Currently, there are two main methods for growing nanorod electrodes. One method involves spin coating glass or filling nanorod gaps with silica for planarization before electrode growth [29,72,75,103], while the other method involves directly transferring graphene as a transparent conductive layer, followed by metal electrode sputtering [28,38,74,104]. Regardless of the method used, it undoubtedly increases production costs, which is a major reason why nanorod structures are difficult to apply in actual production.

## 5. Challenges and Prospects

μLED undoubtedly represents a new display technology for the future; yet, true commercialization remains unrealized due to issues such as luminous brightness, full colorization, and mass transfer. The application of nanomaterials and nanostructures in LEDs presents a pathway to addressing these challenges. Based on the aforementioned studies, nanomaterials such as metal NPs, TiO_2_ NPs, and QDs can assist μLEDs in achieving high-brightness emission and provide new avenues for full colorization. By designing and introducing nanostructures, the optical, electrical, and thermal properties of LED devices can be altered, thereby improving their luminous efficiency, color conversion efficiency, and stability.

For instance, the utilization of nanostructures such as nanorods, nanoholes, and nanorings can enhance LEE, reduce internal reflection and refraction losses, release epitaxial stress, suppress QCSEs, and enhance luminous efficiency. Additionally, nanostructures can reduce the distance between nanomaterials and MQWs, introducing LSP coupling and NRET mechanisms, thereby reducing energy loss and maximizing the enhancement effects of nanomaterials on LED performance.

Although the application of nanomaterials and nanostructures in LEDs can enhance luminous efficiency and CCE, there are also several drawbacks. Metal NPs are prone to oxidation, and if they aggregate in large quantities, they can cause charge energy transfer, leading to overall fluorescence quenching [57]. QDs, as color conversion materials, have poor thermal stability and reliability. Prolonged use in full-color arrays can result in a decrease in CCE [105,106,107].

Similarly, the aforementioned nanostructures also have their own limitations. Nanoring and nanohole structures sacrifice a significant active area, which may lead to a decrease in overall luminous efficiency. Moreover, nanorings and nanorods are discontinuous, posing challenges for the fabrication of metal electrodes. These factors constrain the industrial application of these structures.

As μLED display technology continues to evolve, there are still many core technologies for achieving full-color displays, such as mass transfer [108] and three-color stacking [109,110]. Arrays produced using these methods are more reliable and suitable for large-scale production. The future development of μLED display technology will likely revolve around these two techniques.

Beyond display applications, μLEDs hold enormous potential in the visible light domain due to their ultra-fast light pulses and extremely high modulation bandwidth [111,112,113]. In particular, with the continuous development of 5G and 6G networks in recent years, μLED visible light communication (VLS) is poised to have a significant application market. The nanomaterials and nanostructures discussed in this review can also be applied in μLED VLS to further increase its modulation bandwidth and transmission rate or to realize optical communication at different wavelengths.

## Figures and Tables

**Table 1:** Photoluminescence (PL) decay times of various metal NP samples when they are linked with QDs and placed on top of LEDs. The six samples in the table represent LEDs without QDs and metal NPs, LEDs with QDs, LEDs with QDs and metal NPs with an absorption peak at 420 nm, LEDs with QDs and metal NPs with an absorption peak at 465 nm, LEDs with QDs and metal NPs with an absorption peak at 550 nm, and LEDs with QDs and metal NPs with an absorption peak at 645 nm. [43] Reprinted with permission from ref. [43]. Copyright {2019} Optica Publishing Group.

Sample	NP	QW PL Decay Time (ns)	QD PL Decay Time (ns)
R	–	1.84	–
QD	–	2.21	7.76
QD-NP420	Ag	2.07	5.08
QD-NP465	Ag	1.93	4.18
QD-NP550	Ag	2.01	4.39
QD-NP645	Ag/Au shell	2.14	5.83

## Data Availability

Not applicable.
